# Preparation of Iron-Doped Titania Nanoparticles and Their UV-Blue Light-Shielding Capabilities in Polyurethane

**DOI:** 10.3390/ma15207370

**Published:** 2022-10-21

**Authors:** Regina Baimanova, Fushuai Luo, Mingshu Yang

**Affiliations:** 1 Key Laboratory of Engineering Plastics, Institute of Chemistry, Chinese Academy of Sciences, Beijing 100190, China; 2 University of Chinese Academy of Sciences, Beijing 100049, China

**Keywords:** iron-doped titania nanoparticles, UV-blue light-shielding, photocatalytic activity, nanocomposites, polyurethane

## Abstract

It is well known that ultraviolet (UV) and blue light cause a series of health problems and damages to polymer materials. Therefore, there are increasing demands for UV-blue light-shielding. Herein, a new type of iron-doped titania (Fe-TiO_2_) nanoparticle was synthesized. Fe-TiO_2_ nanoparticles with small particle size (ca. 10 nm) are composed of anatase and brookite. The iron element is incorporated into the lattice of titania and forms a hematite phase (α-Fe_2_O_3_). The iron doping imparted full-band UV and blue light absorption to Fe-TiO_2_ nanoparticles, and greatly suppressed the photocatalytic activity. The prepared Fe-TiO_2_/polyurethane (PU) films exhibited prominent UV-blue light-shielding performance and high transparency, which showed great potential in light-shielding fields.

## 1. Introduction

Harms to human health imposed by ultraviolet (UV) light, e. g. the damages to skin and DNA, have been investigated in detail [1,2]. Meanwhile, UV light gives rise to the photoaging and results in the deterioration of performance of organic materials [3,4,5]. On the other hand, blue light (400–500 nm) which widely exists in the sunlight and electronic devices [6,7], has been confirmed to cause a series of health problems. For instance, it causes irreversible damage to the retina and inhibits formation of melatonin [8,9,10]. As a result, there is a critical demand for the development of effective UV-blue light-shielding materials to protect human health and organic materials.

Nowadays, organic UV absorbers and yellow dyes are widely used as UV-blue light-shielding agents in transparent polymer materials. However, the poor photo- and/or thermal-stability and weak solvent extraction resistance shorten the service life [11]. Titania (TiO_2_) nanoparticles with excellent UV absorption, photo- and thermal-stability and nontoxicity, have been considered as a promising light-shielding materials [12,13,14]. Though some progress has been made in the field of TiO_2_-based UV light-shielding materials, several issues remain challenges. Due to the wide bandgap (3.0–3.2 eV), TiO_2_ nanoparticles have limited UV absorption range (usually <380 nm) [15]. Moreover, high photocatalytic activity would accelerate the photodegradation of polymer materials. Fortunately, element doping, including with Fe and N, can reduce the bandgap and photocatalysis of TiO_2_ nanoparticles in the meantime [12,16,17], which makes TiO_2_ nanoparticles suitable as UV-blue light-shielding agents. Particularly, Fe^3+^ owns an approximate atom radius with Ti^4+^, which would greatly facilitate its doping into the bulk lattice of TiO_2_ [18]. Furthermore, the negative impacts on the transparency of polymer should be considered owing to the high refractive index and scattering effect of TiO_2_ nanoparticles. An alternative option is to decrease the particle size according to the Rayleigh scattering theory, which indicates that nanoparticles with diameters smaller than 40 nm have negligible effects on the transparency of polymer nanocomposites [19,20].

Herein, novel iron-doped titania (Fe-TiO_2_) nanoparticles with small size (about 10 nm) were synthesized by a simple solution method. The crystal and doping structures of Fe-TiO_2_ were carefully characterized. Fe-TiO_2_ nanoparticles had full-band UV and blue light absorption and restricted photocatalytic activity, which showed great potential as UV-blue light-shielding agents in transparent polymer materials. The prepared Fe-TiO_2_/polyurethane (PU) films exhibited excellent UV-blue light-shielding performance.

## 2. Materials and Methods

### 2.1. Synthesis of Fe-TiO_2_ Nanoparticles

Firstly, Ti(IV) precursor solution was prepared by dissolving 1.0 mL TiCl_4_ into 1.5 mL HCl solution (36.5–38.0 wt%) and then diluted by 20 mL distilled water. In another flask, 1.0 g FeCl_3_ in 300 mL water was heated to 100 °C to get a dark red solution, and then Ti(IV) precursor solution was poured in. The reaction remained at 100 °C for 4 h and stopped. Fe-TiO_2_ nanoparticles were precipitated by adding acetone, and then washed by water and acetone. Finally, Fe-TiO_2_ nanoparticles were dispersed into ethanol solution. Pure TiO_2_ nanoparticles were prepared by the same method, except for the addition of FeCl_3._

### 2.2. Preparation of Fe-TiO_2_/PU Nanocomposites Films

The polyurethane was prepared by a commonly used method [21]. PU was completely dissolved in DMF at 80 °C, then Fe-TiO_2_/ethanol solution was added and stirred for 2 h at 80 °C. The polymer solution was poured into the mold and solvent was evaporated in oven for 12 h at 60 °C. Finally, films with thickness of ca. 200 μm with a Fe-TiO_2_ content of 0.1, 0.3, 0.5, 0.7, and 1.0 wt% were obtained, respectively.

### 2.3. Characterization

Transmission Electron Microscopy (TEM) images were taken on a JEM-1011 (JEOL, Tokyo, Japan). The crystal composition was identified by X-Ray Diffraction (XRD) on a D/max–2500 diffractometer (Rigaku, Tokyo, Japan) with Cu-Kα (λ = 0.154 nm) radiation. The data was recorded at a scan speed of 8 °/min within 10–70°. X-ray Photoelectron Spectra (XPS) was carried out on an ESCALAB250XI instrument (Thermo Fisher Scientific, Waltham, MA, USA) with an Al Kα X-ray source (E = 1486.6 eV). Ultraviolet Visible (UV-Vis) Diffuse Reflectance Spectroscopy of Fe-TiO_2_ nanoparticles powders was collected on a UV-2600 spectrophotometer (Shimadzu, Tokyo, Japan). The reflectance (R%) was converted into the absorbance (Abs) through Kubelka–Munk conversion. UV-Vis Transmission Spectra of PU nanocomposite films were collected with a scanning rate of 480 nm/min from 200 to 800 nm on Lambda 35 UV-vis Spectrometer (Perkin Elmer, Waltham, MA, USA).

## 3. Results and Discussion

### 3.1. Structure of Fe-TiO_2_ Nanoparticles

**Figure 1:**
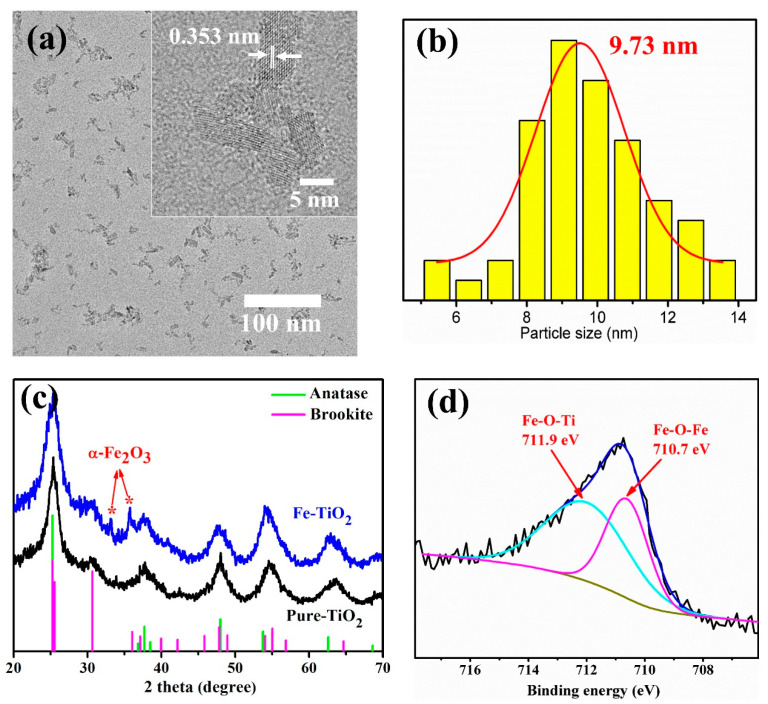
(**a**) TEM, high-resolution TEM image (inset), (**b**) distribution of particle size and (**c**) XRD spectra of Fe-TiO_2_ and Pure-TiO_2_ nanoparticles. (**d**) XPS fine spectrum of Fe 2p_3/2_.

The morphology and structure of Fe-TiO_2_ nanoparticles were characterized. As shown in Figure 1a,b, Fe-TiO_2_ nanoparticles with an average size of ca. 10 nm were in the shape of short nanorods. In Figure 1c, XRD showed that Fe-TiO_2_ and Pure-TiO_2_ nanoparticles consisted of anatase (PDF#71-1167) as a major crystal phase and brookite (PDF#76-1937) as a secondary crystal phase. The interplanar spacing of 0.353 nm (Figure 1a inset) corresponded to the (101) crystal plane of anatase, which was consistent with XRD results. It is worth noting that two diffraction peaks appeared in 33.2° and 35.6°, which were ascribed to (104) and (110) crystal planes of hematite (α-Fe_2_O_3_, PDF#33-0664), respectively. XPS fine spectrum of Fe 2p_3/2_ (Figure 1d) indicated that Fe element existed in the forms of Fe-O-Ti and Fe-O-Fe structure. The XRD and XPS results demonstrated that Fe element successfully doped into the lattice of TiO_2_ and generated α-Fe_2_O_3_ phase in Fe-TiO_2_ nanoparticles.

### 3.2. Light Absorption and Photocatalysis of Fe-TiO_2_ Nanoparticles

**Figure 2:**
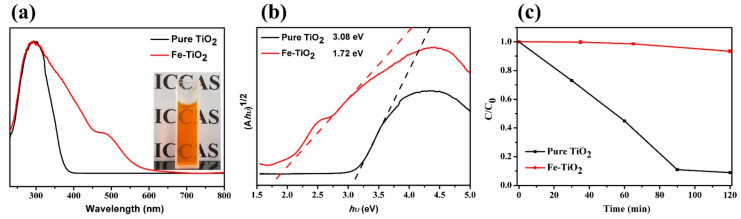
(**a**) UV-vis DRS spectra, (**b**) corresponding (Ah*v*)^1/2^~h*v* curves and (**c**) photocatalysis curves of pure TiO_2_ and Fe-TiO_2_ nanoparticles.

The light absorption property of Fe-TiO_2_ nanoparticles was tested and compared with neat TiO_2_ nanoparticles, which were synthesized by the same procedure, except for the addition of FeCl_3_. As shown in Figure 2a, Fe-TiO_2_ nanoparticles in water solution exhibited orange color and high transparency, which was attributed to the ultrasmall particle size and good stability in water. UV-vis diffuse reflectance spectrum (DRS) showed that Fe-TiO_2_ nanoparticles possessed the full-band UV and blue light (400–500 nm) absorption capability, while the neat TiO_2_ nanoparticles could only absorb UV light below 380 nm. Then the optical bandgaps were extrapolated from the (Ah*v*)^1/2^~h*v* curves, as shown in Figure 2b. The bandgap decreased from 3.08 eV of neat TiO_2_ to 1.72 eV of Fe-TiO_2_ nanoparticles, which suggested the redshift of the absorption edge. These great differences indicated the iron-doping effect can enhance the UV-absorbing capacity and extend the light absorption range to visible light. The excellent UV-blue light absorbing capability made Fe-TiO_2_ nanoparticles suitable as UV-blue light-shielding agents.

Moreover, the photocatalytic activity of Fe-TiO_2_ nanoparticles was measured via catalyzing the photodegradation of methyl orange (MO). As shown in Figure 2c, pure TiO_2_ nanoparticles showed very high photocatalysis efficiency, just like common anatase TiO_2_. However, FeTiO_2_ nanoparticles had a much lower photocatalysis rate, which demonstrated the restricted photocatalytic activity. As we know, photogenerated electron–hole pairs of TiO_2_ induced by UV light can migrate to the surface to initiate the redox reactions, which accounts for the photocatalysis of TiO_2_. In Fe-TiO_2_ nanoparticles, the electron–hole pairs could be trapped by Fe-O-Ti and/or Fe-O-Fe structure and then recombined, which therefore restrained the photocatalysis. The low photocatalytic activity could reduce the risks of the damage to polymer caused by the photocatalysis of TiO_2_.

### 3.3. UV-Blue Light-Shielding Performance of Fe-TiO_2_/PU Films

**Figure 3:**
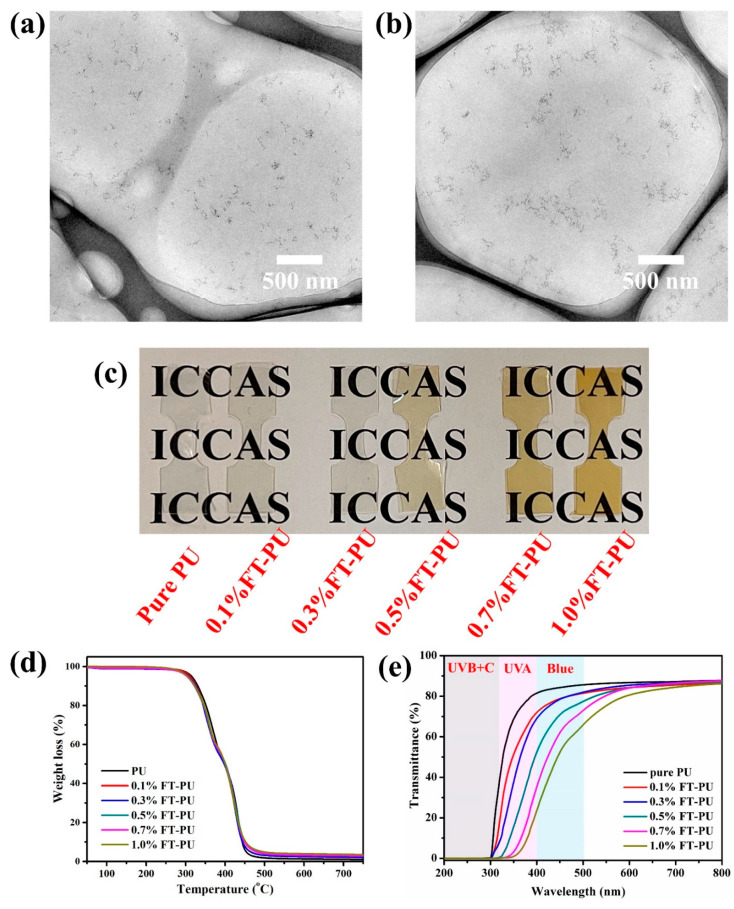
TEM images of ultrathin section of (**a**) 0.5%FT-PU and (**b**) 1.0%FT-PU films. (**c**) Photograph of neat PU and FT-PU films. (**d**) TGA curves of PU nanocomposite films. (**e**) UV-vis transmission spectra of neat PU and PU-FT nanocomposites.

Fe-TiO_2_ nanoparticles were incorporated into polyurethane to prepare Fe-TiO_2_/PU (FT-PU) films with particle content of 0.1, 0.3, 0.5, 0.7, and 1.0 wt%, respectively. Figure 3c showed pure PU and FT-PU films with high transparency. As shown in Figure 3a,b, Fe-TiO_2_ nanoparticles were homogeneously dispersed in PU without obvious aggregation. The small particle size (about 10 nm) and good dispersion minimized the scattering effect of the nanoparticles, which accounted for the high transparency of FT-PU films. As shown in Figure 3d, TGA analysis indicated that the incorporation of Fe-TiO2 nanoparticles has negligible effect on the thermal stability of PU.

The UV-blue light-shielding properties and visible transparency of FT-PU films were characterized by UV-vis transmission spectroscopy. As shown in Figure 3e, neat PU film had good transparency in visible light regions but very limited UV absorption, which was inappropriate for UV-blue light-shielding materials. However, the addition of Fe-TiO_2_ nanoparticles imparted enhanced UV absorption capability to PU films, and remained highly transparent in visible light. With the content of Fe-TiO_2_ nanoparticles increased (from 0.1 wt% to 1.0 wt%), the absorption edge of FT-PU films redshifted, which significantly improved the UV light-shielding property of PU films. It is worth noting that the transmittance in the blue light region (400–500 nm) of FT-PU films decreased a lot, which suggested its great potential as a kind of blue light-shielding material.

The UV-blue light-shielding performances were further evaluated by quantitative calculation of the UVA, UVB, blue blocking rates of PU and FT-PU films. The UVA, UVB, blue shielding rates were calculated by the equations: [22]

(1) $$\mathrm{UV}-A\;\mathrm{blocking}\;\mathrm{rate}\;(\%)=100\;-\;\frac{\int_{320}^{400}T\left(\lambda \right)\;d\lambda }{\int_{320}^{400}\;d\lambda }\;(\%)$$

(2) $$\mathrm{UV}-B\;\mathrm{blocking}\;\mathrm{rate}\;(\%)=100\;-\;\frac{\int_{280}^{320}T\left(\lambda \right)\;d\lambda }{\int_{280}^{320}\;d\lambda }\;(\%)$$

(3) $$\mathrm{Blue}\;\mathrm{blocking}\;\mathrm{rate}\;(\%)=100\;-\;\frac{\int_{400}^{500}T\left(\lambda \right)\;d\lambda }{\int_{400}^{500}\;d\lambda }\;(\%)$$

where *T(λ)* is the transmittance of PU and FT-PU films, and *λ* is the wavelength (nm). As shown in Table 1, UVA, UVB, blue shielding rates of neat PU film were 30.92%, 89.84%, 15.92%, respectively, indicating poor UV-blue light-shielding properties. With increasing content of Fe-TiO_2_ nanoparticles, the UV-blue blocking rates increased. To be specific, UVB blocking rates of all FT-PU films were higher than 96%, indicating that UVB light could be completely shielded. Meanwhile, the UVA blocking rate was improved from 30.92% of neat PU to 93.91% of 1.0% FT-PU film, which showed excellent UV-shielding performance of FT-PU films. Moreover, blue light was blocked more than 40% and 50% when the content of Fe-TiO_2_ nanoparticles was higher than 0.7% and 1.0%, respectively, which demonstrated the high-efficiency blue light-shielding performance. Furthermore, the visible light transparency of FT-PU films should be concerned. Due to the blue light-shielding property, we calculated the average transmittance in 500–800 nm. The average transmittance slightly decreased from 86.92% of neat PU to 80.93% of 1.0% FT-PU films, which guaranteed the application value in the field of transparent optical materials.

## 4. Conclusions

In summary, we synthesized a kind of novel iron-doped titania nanoparticles with small particle size (10 nm), in which Fe element was in the forms of Fe-O-Ti and Fe-O-Fe structures. The iron-doping endowed Fe-TiO_2_ nanoparticles with full-band UV and blue light absorption properties and restricted photocatalytic activity. The prepared Fe-TiO_2_/polyurethane films exhibited improved UV-blue light-shielding performance with the increasing content of Fe-TiO_2_ nanoparticles. 1.0% FT-PU film showed more than 99%, 93%, 50% of UVB, UVA, blue light-shielding rate and 80% of visible transparency, respectively. The high-performance UV-blue light-shielding properties and good transparency of Fe-TiO_2_/polyurethane films showed great application value in transparent light-shielding materials.

## Figures and Tables

**Table 1:** UV-blue light-shielding rate of PU and FT-PU nanocomposites films.

Sample	UVB (280–320 nm)	UVA (320–400 nm)	Blue (400–500 nm)	Ave-T (500–800 nm)
PU	89.84	30.92	15.92	86.92
0.1%FT-PU	96.56	46.50	21.92	84.66
0.3%FT-PU	98.00	56.59	22.58	85.71
0.5%FT-PU	99.91	76.13	31.32	84.47
0.7%FT-PU	99.97	88.98	41.02	84.12
1.0%FT-PU	99.93	93.91	50.73	80.93

## Data Availability

The data presented in this study are available on reasonable request from the corresponding author.
